# Stokes Spectropolarimetry Applied to Measure Circular Birefringence Dispersion of Aqueous Solutions of Sugars

**DOI:** 10.1002/chir.70047

**Published:** 2025-07-08

**Authors:** Ruan Lucas Sousa Lima, Eric Santos da Silva, Paulo Trindade Araujo, Newton Martins Barbosa Neto

**Affiliations:** ^1^ Institute of Natural Sciences Federal University of Pará Belém Pará Brazil; ^2^ Department of Astronomy and Physics The University of Alabama Tuscaloosa Alabama USA

**Keywords:** circular birefringence, optical activity, optical rotatory dispersion, spectropolarimetry

## Abstract

Circular birefringence (CB) is defined as the difference in refractive index for opposite circular polarization states and has played a crucial role in the development of stereochemistry and the concept of chirality. It manifests experimentally as optical rotatory dispersion (ORD), that is, the wavelength‐dependent optical rotation of the plane of light polarization. However, most methods for probing ORD rely on analyzing transmitted light asymmetry at single wavelengths (usually the sodium D‐line at 589 nm) with linear polarizers, which cannot discern between unpolarized and circularly polarized light, limiting the apparatus to analyze a single phenomenon. Here we showcase the use of Stokes spectropolarimetry (SSP), a versatile and cost‐effective technique, to probe ORD of circularly birefringent materials. This technique allows complete analysis of the dispersive changes in polarization caused by anisotropic media, portraying a versatile experimental framework to study different types of optical anisotropies with a single spectropolarimeter. Here, aqueous solutions of chiral sucrose, fructose, and their mixtures are investigated. The ORD acquired verify that the optical rotation is proportional to the concentration of the chiral species and follows an inverse proportion with wavelength. As a case study, we show via SSP that ORD at 589 nm (D‐line of sodium) is in good agreement with literature (+63.5° ± 1.4° mL g^−1^ dm^−1^ for sucrose and −83.7° ± 2.0° mL g^−1^ dm^−1^ for fructose).

## Graphical abstract

This work demonstrates the use of Stokes spectropolarimetry (SSP) to investigate optical rotatory dispersion (ORD), or circular birefringence (CB), of chiral sugar solutions. The results confirm the relationship between concentration and optical rotation, the dependence on wavelength, and the versatility of SSP to characterize optical anisotropies.

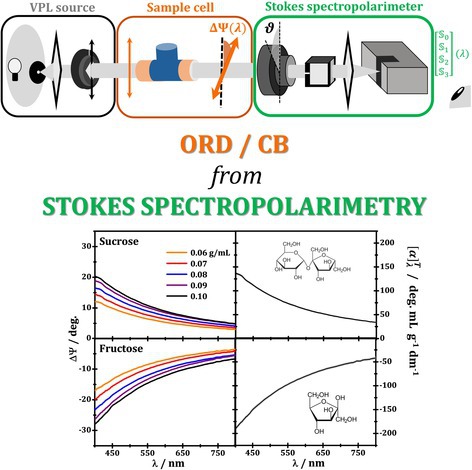

## 1 Introduction

Among many forms of resonant and non‐resonant refraction‐based optical anisotropies a medium can exhibit [1, 2, 3], circular birefringence (CB) has played a crucial role in the development of stereochemistry and the concept of *chirality* [4, 5]. It manifests experimentally as optical rotatory dispersion (ORD), a ubiquitous practice for determining the wavelength‐resolved rotation of the plane of polarization [1, 5, 6, 7]. This phenomenon was first observed in quartz crystals by Arago in 1811, and then in 1815, Biot observed it in liquid solutions of turpentine oil and camphor in alcohol [8, 9]. The parameter that quantifies the intrinsic rotatory power of sample at a given wavelength in each environment is called *specific optical rotation* (SOR). It measures the optical rotation per unit length per unit concentration, being represented by ${\left[\alpha \right]}_{\lambda }^{T}$, where λ is the wavelength being analyzed and T is the temperature. If SOR is resolved over wavelength, the *specific optical rotatory dispersion* (SORD) is obtained [4, 7, 10]. Since it is an intrinsic property of materials, it is often used to quantify the concentration of species in liquids, for example, the amount of sugar in wines [1]. Regardless of the quantity measured to characterize this type of optical activity, the physical origin remains the same.

Physically, CB arises from the difference in refractive indices for right‐ (clockwise) and left‐handed (counterclockwise) circularly polarized light, with directions being defined by the detector's perspective [1, 4]. For further details on the phenomenological description of CB, see Section S1. As first explained by Fresnel in 1825 [8], when a linearly polarized (or *plane‐polarized*) light crosses a circularly birefringent medium it is rotated either clockwise or counterclockwise, depending on the sign of birefringence. A medium that causes a clockwise (or right‐handed) rotation of plane polarized light is called *dextrorotatory*, whereas a counterclockwise (or left‐handed) rotation is caused by a *levorotatory* medium. For a medium to be *dextro‐* or *levorotatory*, it must present some kind of circular symmetry breaking, which is observed whenever such medium present either natural or induced *chirality* [4].

In terms of symmetry, an object is called *chiral* if it is non‐superimposable on its mirror image by translation or rotation [4, 11]. Many organic molecules possess chirality, for instance, saccharides, like D‐glucose, and amino acids, like L‐tryptophan, which are respectively *dextrorotatory* (right‐handed) and *levorotatory* (left‐handed) [10, 12, 13]. Probing chirality is, therefore, a strategy to delve deeper into nuances of biochemical mechanisms, as well as to provide optical insights into other induced symmetry breaking, with magnetic fields, for example [10, 14, 15]. In this context, simple commercial sugars, such as sucrose and fructose, which present opposite chirality signs, are appropriate candidates for reference materials to test novel new experimental setups [13, 16, 17, 18].

Specifically, while fructose is a levorotatory monosaccharide with SOR of ${\left[\alpha \right]}_{D}=-92^{{}^{\circ}}\;\mathrm{mL}\;g^{-1}\;dm^{-1}$, sucrose is a dextrorotatory disaccharide formed by the combination of a glucose and fructose molecules, with SOR of ${\left[\alpha \right]}_{D}=+66.5^{{}^{\circ}}\;\mathrm{mL}\;g^{-1}\;dm^{-1}$ [10, 16]. Interestingly, adding acid to an aqueous solution of sucrose causes its hydrolyzation, which produces an even mixture of glucose and fructose, also known as *inverted sugar*, which has a SOR of ${\left[\alpha \right]}_{D}=-22^{{}^{\circ}}\;\mathrm{mL}\;g^{-1}dm^{-1}$. The sign change (inversion) in optical rotation with hydrolyzation, meaning a transition from dextrorotatory to levorotatory, gives the name of the mixture [19].

Many methods for probing ORD have been proposed [9, 20, 21, 22, 23], the most widespread versions being variations of Biot's polarimeter, where a polarizer is rotated until null transmitted light intensity is reached [8, 9, 24]. However, most of these methods rely on analyzing transmitted light asymmetry with linear polarizers, which cannot discern between unpolarized and circularly polarized light. While these methods might be enough for standalone ORD measurements (i.e., for apparatuses specifically built to measure ORD only), if other types of anisotropy are also of interest, a whole different equipment would be needed. In this sense, having a setup capable of characterizing many different forms of anisotropy with the least number of adjustments in the experimental apparatus is highly desirable. In addition, most methods rely on single‐wavelength characterization, usually of sodium D‐line at 589 nm (hence, the D in ${\left[\alpha \right]}_{D}$ references) as the standard reference for historical reasons [9]. The use of tunable lasers for the characterization of dispersion has been an alternative, but methods of measuring ORD for a broad wavelength range at once with use of charge‐coupled devices (CCDs) attain much more information in a single acquisition than by discretely tuning the wavelength under analysis [20, 25, 26].

Since anisotropic materials are highly sensitive to polarized light, probing how light polarization is modified due to interactions with a medium is an efficient approach to acquire anisotropy data. Besides, acquisition of such anisotropy over a broad wavelength range adds another dimension to the analysis, since every refraction‐based anisotropy is strongly dependent on light's frequency. In this context, Stokes spectropolarimetry (SSP) becomes a versatile framework to study static forms of optical anisotropy [25, 27, 28, 29, 30, 31, 32]. Based on the rotating retarder Fourier analysis method [3], the Stokes parameters of light obtained via SSP are determined over a broad wavelength range. It means that the spectral distributions of Stokes parameters, hence information about degree of polarization and ellipse of polarization, are fully determined for any source of polychromatic light. For example, the technique has been utilized in a previous work to study linear birefringence (LB) of transparent adhesive tapes and other phase shifting elements [27].

The concept of spectropolarimetry was initiated with Biot in 1817, when he reported that the optical rotation is greater for shorter wavelengths, but only 136 years later Carl Djerassi would start to systematically measure wavelength‐resolved optical rotation, that is, ORD [9]. From this point of view, this manuscript adds a modern approach to this long‐standing history. We report on using SSP of transmitted light to study circular birefringence in aqueous solutions of chiral sugars. We do so by measuring their ORD in solutions of single species at different concentrations, as well as mixtures at different proportions. By changing the chiral species (sucrose vs. fructose), or the concentration of each solution (from 0.06 to 0.10 g/mL), or the fractional contribution of each species in a fixed concentration solution (0%, 20%, …, 80%, 100% of fructose from a 0.10 g/mL mixed solution), clear correlations between polarimetric observables and the optical activity of these chiral molecular systems are readily established.

Here, the basic theory behind CB and its connection to Stokes parameters of transmitted light are presented in Section S1, and an approach to acquire CB via Stokes parameters with simple case studies is developed. Although the measurement of either optical rotation or Stokes parameters are not new [3, 5, 22, 33, 34, 35, 36, 37], this approach aims to showcase the simplicity and versatility of probing changes in polarization state caused by anisotropic media using a single spectropolarimeter, in this case applied to the transmitted light by transparent (non‐resonant) chiral media. Correlating and quantifying such effects is of paramount interest to materials science, analytical chemistry, as well as to higher education programs. This includes teaching light polarization to optics students or molecular chirality to chemistry majors, as has been reported extensively with other approaches [12, 17, 18, 24, 35, 38, 39, 40, 41, 42].

## 2 CB From Stokes Parameters Data

Analogously to the LB phenomena [3, 27], one may assume a linear transformation of components in the interaction of light with the birefringent medium, such that

(1a) $$E_{L}^{\prime }=\exp\left(-i\;\Upsilon_{0}/2\right)\;E_{L},$$

(1b) $$E_{R}^{\prime }=\exp\left(+i\;\Upsilon_{0}/2\right)\;E_{R,}$$

where $E_{L}$ ($E_{L}^{\prime })$ and $E_{R}$ ($E_{R}^{\prime })$ are the complex amplitudes of, respectively, left‐handed and right‐handed circular components of incident (transmitted) light, whereas $\Upsilon_{0}$ is the medium's circular retardance, related to circular birefringence according to Equation (S1). Using equations

(2a) $$E_{L}=\frac{1}{\sqrt{2}}\;\left(E_{x}+i\;E_{y}\right),$$

(2b) $$E_{R}=\frac{1}{\sqrt{2}}\;\left(E_{x}-i\;E_{y}\right),$$

Equations (1a) and (1b) can be rewritten as a complex linear combination of the *x*–*y* cartesian components [43, 44, 45]. Therefore, the transformation of cartesian amplitudes due to a circular birefringent medium is described as

(3a) $$E_{x}^{\prime }=\cos\left(\Upsilon_{0}/2\right)\;E_{x}+\sin\left(\Upsilon_{0}/2\right)\;E_{y},$$

(3b) $$E_{y}^{\prime }=-\sin\left(\Upsilon_{0}/2\right)\;E_{x}+\cos\left(\Upsilon_{0}/2\right)\;E_{y}$$

By combining Equations (3a) and (3b) with the definition of Stokes parameters in terms of complex amplitudes, given by [3].

(4a) $$S_{0}=E_{x}^{*}E_{x}+E_{y}^{*}E_{y},$$

(4b) $$S_{1}=E_{x}^{*}E_{x}-E_{y}^{*}E_{y},$$

(4c) $$S_{2}=E_{x}^{*}E_{y}+E_{y}^{*}E_{x},$$

(4d) $$S_{3}=i\left(E_{x}^{*}E_{y}-E_{y}^{*}E_{x}\right),$$

the linear transformation of Stokes parameters caused by the medium is obtained as

(5a) $$S_{0}^{\prime }=S_{0},$$

(5b) $$S_{1}^{\prime }=\cos\Upsilon_{0}S_{1}+\sin\Upsilon_{0}S_{2},$$

(5c) $$S_{2}^{\prime }=-\sin\Upsilon_{0}S_{1}+\cos\Upsilon_{0}S_{2},$$

(5d) $$S_{3}^{\prime }=S_{3}$$

In one hand, Equation (5a) shows that when light crosses a circularly birefringent medium the total intensity is not attenuated. On the other hand, Equation (5d) shows that the amount of circular polarization, or the ellipticity, remains unchanged. The linear relation between the Stokes parameter for the emerging and incoming lights can be described by a matrix associated with the birefringent medium which promoted the change in polarization state. The *circular birefringence Mueller matrix* is then given by

(6) $$M_{\mathrm{CB}}\left(\Upsilon_{0}\right)=\left[\begin{array}{c} 1\\ 0\\ \begin{array}{c} 0\\ 0 \end{array} \end{array}\;\begin{array}{cc} \begin{array}{c} 0\\ \cos\Upsilon_{0}\\ \begin{array}{c} -\sin\Upsilon_{0}\\ 0 \end{array} \end{array} & \begin{array}{cc} \begin{array}{c} 0\\ \sin\Upsilon_{0}\\ \begin{array}{c} \cos\Upsilon_{0}\\ 0 \end{array} \end{array} & \begin{array}{c} 0\\ 0\\ \begin{array}{c} 0\\ 1 \end{array} \end{array} \end{array} \end{array}\right]$$

Note the matrix in Equation (6) conveys that the net effect of a circularly birefringent medium is the same as of a rotator which rotates the polarization ellipse clockwise by an angle of $\Upsilon_{0}/2$ [3]. Such optical rotation can be directly assessed by means of elliptical inclination, given by [3, 36, 37].

(7) $$\tan\left(2\Psi^{\prime }\right)=S_{2}^{\prime }/S_{1}^{\prime }$$

By substituting Equations (5b) and (5c) into Equation (7), it is obtained that

(8) $$\begin{array}{c} \frac{S_{2}^{\prime }}{S_{1}^{\prime }}=\frac{-\sin\Upsilon_{0}\;S_{1}+\cos\Upsilon_{0}\;S_{2}}{\cos\Upsilon_{0}\;S_{1}+\sin\Upsilon_{0}\;S_{2}}=\frac{\tan\left(2\Psi \right)-\tan\left(\Upsilon_{0}\right)}{1+\tan\left(2\Psi \right)\tan\left(\Upsilon_{0}\right)}\\ \;=\tan\left(2\Psi -\Upsilon_{0}\right)=\tan\left(\Psi^{\prime }\right), \end{array}$$

with

(9) $$\Psi^{\prime }=\Psi -\Upsilon_{0}/2\;,$$

where by definition, $0\leq \Psi^{\prime }<180^{{}^{\circ}}$ relative to the horizontal direction, increasing in the counterclockwise direction [3]. Hence, a positive circular retardance causes the polarization ellipse to rotate clockwise by decreasing the inclination angle from the incident beam's reference orientation Ψ (e.g., $\Psi =90^{{}^{\circ}}$ for vertically polarized light).

In this context, to follow the sign convention where a clockwise rotation is said to be a positive rotation [1], the optical rotation generated by CB should be defined as

(10) $$\Delta \Psi =-\left(\Psi^{\prime }-\Psi \right)=\Upsilon_{0}/2$$

Notice that by doing so, a positive (negative) circular retardance $\Upsilon_{0}$, meaning that the right‐handed circular component has gained (lost) a phase relative to the left‐handed one as given by Equations (1a) and (1b) due to a higher (lower) propagation speed in that medium, yields a clockwise optical rotation, ΔΨ>0 (counterclockwise, ΔΨ<0).

Hence, an optically active sample causes a rotation of the ellipse of polarization by an angle half the phase difference between the right and left‐handed circular components of light. It is worth highlighting that the direction and magnitude of the rotation angle is related to the difference in refringence, which defines the term *birefringence*, according to [1, 3].

(11) $$\Delta \Psi =\frac{\mathrm{\pi L}}{\lambda }\;\left(n_{L}-n_{R}\right),$$

where $n_{L}$ and $n_{R}$ are respectively the index of refraction for the left‐ and right‐handed circularly polarized light, L is the optical path of the medium, and λ is the wavelength.

Equation (11) shows that if the index of refraction for left‐handed light is the greatest, then that component is slowed down, and thus a clockwise rotation occurs, as illustrated in Figure S1. In this case, the right‐handed (clockwise) refractive index could also be referred as the *fast axis*, in analogy to the LB counterpart [27]. The opposite assumption yields the opposite conclusion. In the former case, we would have a dextrorotatory sample (clockwise), and in the latter case, a levorotatory sample (counterclockwise) [1, 3]. In addition, since the phase difference ($\Upsilon_{0})$ is associated with different optical paths ($n_{i}L,\;i=L,R)$, it becomes clear from this derivation that circular birefringence (one distinct index of refraction for each orthogonal circular polarization state) generates a rotation in the polarization ellipse, without changing ellipticity, as predicted by Equation (5d).

## 3 Experimental Methodology

**FIGURE 1:**
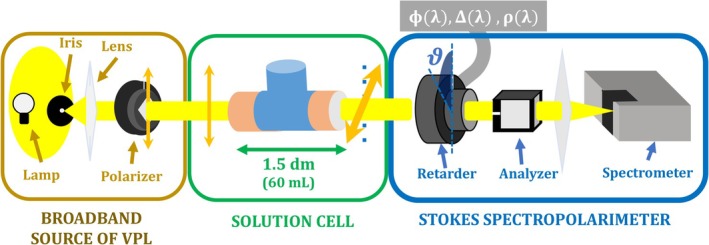
Schematic representation of elements and parts of the apparatus to measure the optical activity of sugar solutions. From left to right: the yellow box marks a broadband source of vertically polarized light (VPL), where a halogen lamp light is collimated and polarized vertically by a calcite polarizer; the green box entails the home‐built solution cell, through which light is transmitted, and its polarization state is transformed; the blue box highlights the Stokes spectropolarimeter, which acquires the spectral distribution of Stokes parameters of light. It encompasses a rotating retarder followed by a fixed horizontal polarizer, and the spectral signal is detected by a portable spectrometer. The retarder is rotated counterclockwise relative to the detector by an angle of ϑ in steps of 10° until a full rotation is completed. As described in reference [25], its retardance spectrum $\Phi \left(\lambda \right)$, the true fast axis tilt‐from‐mark dispersion $\Delta \left(\lambda \right)$, and its optical rotation dispersion $\rho \left(\lambda \right)$ are calibrated and used to calculate artifact‐free Stokes parameters spectra.

Figure 1 shows the experimental apparatus divided into three parts. The first part (yellow box) depicts the broadband source of vertically polarized light, where the thermal emission of a 105 W halogen lamp is collimated and polarized vertically by a calcite crystal polarizer (Glan–Thompson calcite polarizer, Thorlabs). This part sets a reference signal that will be modified upon interaction with the solutions, which will be sitting in a home‐built *solution cell*, shown in the green box, containing approximately 60 mL of sample solution, and 15 cm of optical path length. The *optical activity* is detected as a change in the Stokes parameters of light, or equivalently, in the ellipses of polarization. The Stokes spectropolarimeter, highlighted in the blue box, is the same described in previous work [27], which we briefly summarize below.

Briefly, the Stokes spectropolarimeter is based on the Fourier analysis of the signal detected via the rotating‐retarder method [3, 25]. In this method, the beam of interest (in our case, the beam transmitted through a sugar solution) passes through a rotating quarter‐wave retarder (achromatic over 400–800 nm, Thorlabs, AQWP05M‐600) and a fixed horizontal polarizer (uncoated Glan‐Laser Calcite Polarizer, Thorlabs, GL10), and then it is spectrally resolved by a portable spectrometer (Ocean Optics, USB2000). By azimuthally rotating the retarder, the detected intensity spectrum is modulated harmonically as a truncated Fourier series [3]. The amplitudes of certain harmonic frequencies at each wavelength are directly proportional to the Stokes parameters of the incoming beam, allowing their spectral acquisition [3].

Note that the actual relationship between amplitude parameters and Stokes parameters strongly depends on the nature and quality of the optical elements utilized. In fact, if ideal optical elements could be considered (e.g., a waveplate retarder having an exact quarter‐wave retardance), standard relations would be used as described elsewhere [3, 25]. Alternatively, the use of an imperfect achromatic retarder, for example, could give rise to spectral artifacts, as addressed in a previous work [25]. Such imperfections arise from small manufacturing azimuthal misalignments between the double birefringent crystal assembly necessary to give the achromaticity of typical commercially available achromatic retarders. Those artifacts are eliminated by using the biplate model, derived in reference [25], for Stokes parameters calculation from the experimental amplitude parameters. By taking intrinsic properties from the retarder into account, namely its retardance spectrum $\Phi \left(\lambda \right)$, its true fast axis tilt‐from‐mark dispersion $\Delta \left(\lambda \right)$, and its equivalent optical rotation dispersion $\rho \left(\lambda \right)$, artifact‐free Stokes parameters spectra are determined and subsequently used to calculate optical rotation dispersions discussed here.

The experimental demonstration of CB was performed by producing five aqueous solutions of sucrose (dextrorotatory) and fructose (levorotatory) at different concentrations, ranging from 0.06 to 0.10 g/mL, in steps of 0.01 g/mL, and measuring the SORD of each sample. Mixed solutions with different volumetric fractions of sucrose and fructose (from 0% to 100% fructose, in steps of 20%) were also measured. In this case, solutions of sucrose and fructose were prepared at a concentration of 0.10 g/mL and then mixed following the desired volumetric proportion. With that, the concentration of the mix solution was assured fixed at 0.10 g/mL, that is, the mass of sucrose plus fructose per unit volume of solution (i.e., 60 mL). This analysis evaluates the competing effect of each chiral species on the mixture's SORD and determines the proportion at which opposing optical rotations canceled each other.

The solutions with pure chirality (sucrose or fructose) were prepared by weighing the appropriate mass (from 3.6 g up to 6.0 g) in a precision balance and diluting them in water until we obtained 60 mL solutions; the approximate capacity of the solution cell. The mixture solutions with distinct chiral species (sucrose and fructose) were prepared by mixing solutions of sucrose and fructose with the same concentration (0.10 g/mL, for stronger effect) at the appropriate volumetric fraction of the final volume (60 mL). To obtain the reference signal, the experiments started with only water in the cell, labeled as a concentration of 0.00 g/mL. Thus, vertically polarized light was transmitted through the 1.5 dm cell, and the signal was acquired according to the rotating‐waveplate polarimetry. The reference solution was then replaced by the prepared sugar solution, until all solutions were tested.

## 4 Results

### 4.1 CB Versus Concentration

**FIGURE 2:**
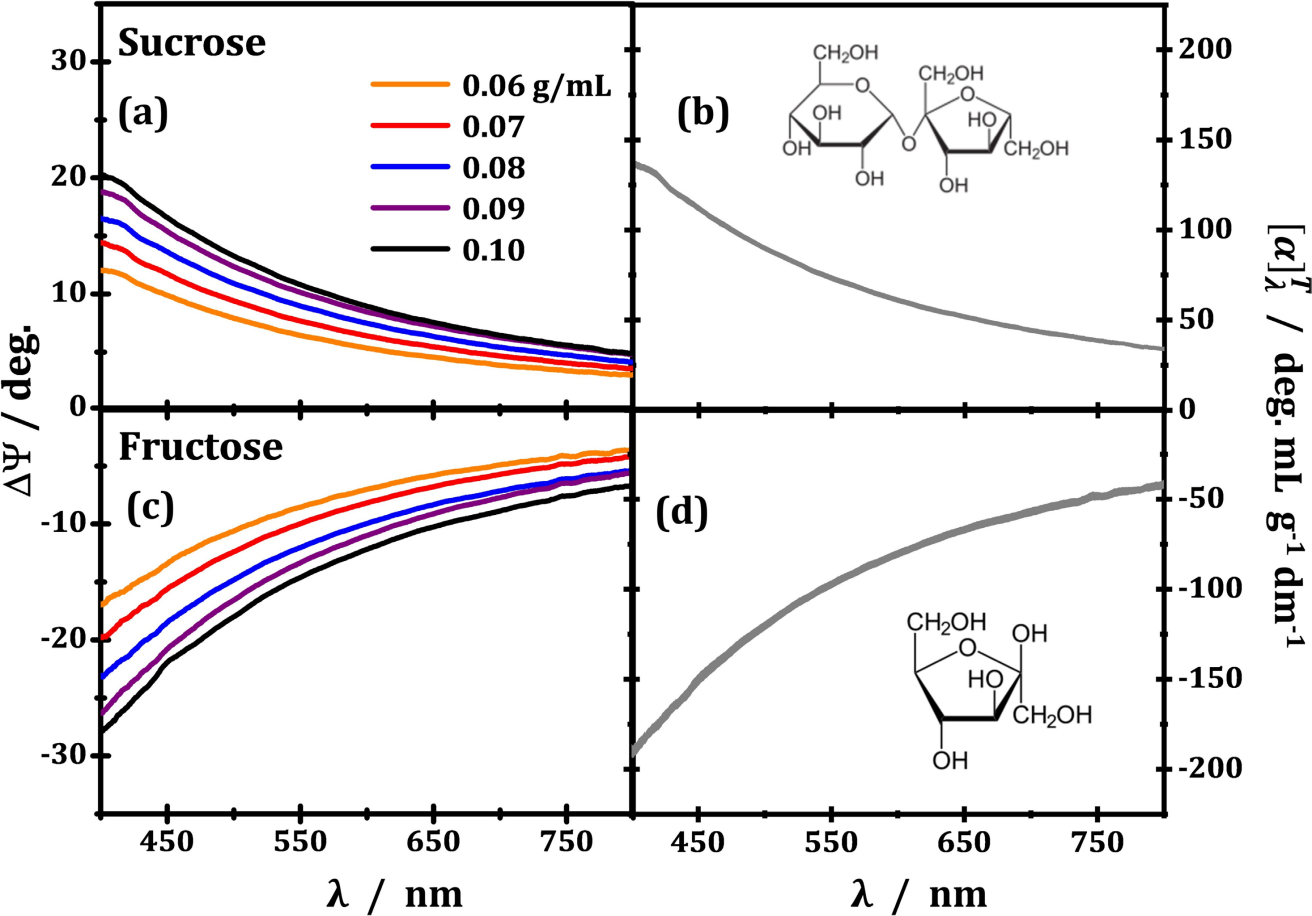
Experimental results for optical rotation (a, c) and specific optical rotation (b, d) dispersions from aqueous solutions of sucrose (a, b) and fructose (c, d) at five different concentrations. The SORD results in panels (b) and (d) have line thickness representing error bar size, given by the standard deviations from the mean calculated from the five optical rotation curves of each chiral species shown in panels (a) and (c). The inset shows the molecular structure of sucrose and fructose in their respective panels.

Here, for simplicity, we present the results for optical rotation ΔΨ without plotting $S_{1}$ and $S_{2}$ dispersions. Note that these quantities are related by Equations (7) and (10). Figure 2a,c displays the wavelength‐dependence of optical rotation for sucrose (positive values) and fructose (negative values) respectively at different concentrations. The dispersions for both chiral species follow an inverse proportionality with wavelength, as well as the linear increase with concentration. The linear trend of optical rotation at the reference λ=589nm (sodium D‐line) as a function of the concentration for sucrose and fructose is displayed in Figure S2. Moreover, as previously discussed (see Equation (10)), in order to keep a clockwise rotation positive and facilitate the comparison with the literature, ΔΨ was calculated by subtracting the reference inclination (e.g., $\Psi =90^{{}^{\circ}}$ for vertically polarized light through solvent only) from the elliptical inclination of a given concentration, such that a positive value means a clockwise rotation (dextrorotatory species), and a negative value means a counterclockwise rotation (levorotatory species). Sucrose (D) and fructose (L) show positive and negative optical rotations ΔΨ, respectively, as expected.

Figure 2b,d exhibits the results of SORD for sucrose (positive side) and fructose (negative side). The curves were calculated by dividing ΔΨ dispersions displayed in Figure 2a,c by solution cell's optical path length (1.5 dm) and by their associated concentrations (e.g., the orange curves were divided by 0.06 g/mL) and then taking their mean value and standard deviation curves for each of the species, displayed as gray curves whose line thickness represent error bar size. It is important to note that SORD represents an intrinsic property of the chiral species in each environment. Therefore, it is often used to estimate the purity of certain chiral solutions (e.g., the amount of sugar in wine) [1, 19]. In Figure 2b,d, SORD at 589 nm (D‐line of sodium lamp emission), at room temperature (within 20°C–25°C), was found to be ${\left[\alpha \right]}_{D}^{20{}^{\circ}C}={\left(+63.5\pm 1.4\right)}^{{}^{\circ}}\;\mathrm{mL}\;g^{-1}dm^{-1}$ for sucrose, and ${\left(-83.7\pm 2.0\right)}^{{}^{\circ}}\;\mathrm{mL}\;g^{-1}dm^{-1}$ for fructose. The literature reports values of, respectively, +66.5 and $-92^{{}^{\circ}}\;\mathrm{mL}\;g^{-1}dm^{-1}$ [46], which yield experimental deviations of 4.5% and 9.0% from our results. Although not substantial, we understand that such deviations are associated with poorer quality of our sugar samples, which were acquired commercially in common food departments.

A complementary analysis can be made using the circular retardance dispersion ($\Upsilon_{0}\left(\lambda \right)$) and specific circular retardance dispersion (circular retardance per unit length per unit concentration as a function of wavelength: $\left[\Upsilon_{0}\left(\lambda \right)\right]/\mathrm{CL}$). This perspective is particularly interesting for comparisons with LB phenomena, which gives rise to linear retardance, as studied in previous work [27]. For instance, a linear retardance value of 0.25 waves is what gives a quarter‐wave plate its power to convert linear polarized light into circularly polarized light [1]. In the circular retardance case, regardless of the phase shift caused between circular components of light, the polarization of light is maintained linear, but in different orientations. Further discussion is available in Section S3.

### 4.2 CB Versus Mixture

Here, we analyze the effect of mixing different chiral species in different proportions on the net optical rotation effect. We derive a simple model to describe the equivalent optical effect of mixing solutions of different chiral species, sucrose (S) and fructose (F), as follows. In a solution, due to their specific translation and rotation inside the medium, the optical effect of two optically active mixed solutions (S and F) is fully equivalent to the sequence of their isolated solutions (S then F, or vice versa). So, it is reasonable to assume that

(12) $$\Delta \Psi_{\mathrm{eq}}=\Delta \Psi_{S}+\Delta \Psi_{F},$$

where $\Delta \Psi_{S}$ is the optical rotation created by sucrose, $\Delta \Psi_{F}$, by fructose, and $\Delta \Psi_{\mathrm{eq}}$ is the optical rotation of an equivalent homogeneous system caused by their sequence. It is hypothesized the existence of a cumulative effect of optical rotations created by each chiral species.

Hence, by making use of Equation (S2), we obtain that

(13) $$\left[\alpha_{\mathrm{eq}}\right]\;C_{\mathrm{eq}}\;L=\left[\alpha_{S}\right]\;C_{S}\;L+\left[\alpha_{F}\right]\;C_{F}\;L,$$

where L is the solution cell's optical path length, $C_{S}$ and $C_{F}$ are, respectively, the concentration of sucrose and fructose relative to the mixture total volume, and $C_{\mathrm{eq}}$ is the concentration of the equivalent system, considering the total solute mass (sucrose + fructose) relative to the total volume. Finally, $\left[\alpha_{S}\right]$ and $\left[\alpha_{F}\right]$ are the respective SORs of sucrose and fructose, and $\left[\alpha_{\mathrm{eq}}\right]$ is the SOR of the mixture.

Considering that $M_{T}=M_{S}+M_{F}$ is the total mass of sucrose ($M_{S}$) and fructose ($M_{F}$) and that $V_{T}$ is the total volume of the mixture, then

(14) $$\left[\alpha_{\mathrm{eq}}\right]\frac{M_{T}}{V_{T}}=\left[\alpha_{S}\right]\;\frac{M_{S}}{V_{T}}+\left[\alpha_{F}\right]\;\frac{M_{F}}{V_{T}}$$

Furthermore, let $f_{X}=M_{X}/M_{T}$ (X=S,F) be the fraction of the chiral species X to the mixture volume, with $f_{S}+f_{F}=1$, thus

(15) $$\begin{array}{c} \left[\alpha_{\mathrm{eq}}\right]\;=\left[\alpha_{S}\right]\;f_{S}+\left[\alpha_{F}\right]\;f_{F}\\ =\left[\alpha_{S}\right]\;\left(1-f_{F}\right)+\left[\alpha_{F}\right]\;f_{F} \end{array}$$

(16) $$∴\left[\alpha_{\mathrm{eq}}\right]\;=\left[\alpha_{S}\right]-\left(\left[\alpha_{S}\right]-\left[\alpha_{F}\right]\right)\;f_{F}$$

Therefore, from Equation (16), it is expected that the experimentally obtained SOR dispersion $\left[\alpha_{\mathrm{eq}}\right]=\Delta \Psi_{\mathrm{eq}}/LC_{\mathrm{eq}}$ will vary linearly with the fraction of fructose.

Now, note that from Equations (S2) and (10), the following holds true

(17) $$\Delta \Psi =\frac{\pi }{\lambda }L\;\Delta n={\left[\alpha \right]}_{\lambda }^{T}\;C\;L$$

Equation (17), where λ is in nm and ${\left[\alpha \right]}_{\lambda }^{T}$ is in deg mL g^−1^ dm^−1^, suggests that for solutions it is worth defining the *specific circular birefringence* (birefringence per unit concentration) and estimates it from SORD curves as according to

(18) $$\Delta n/C=\lambda \;{\left[\alpha \right]}_{\lambda }^{T}10^{-8}/180^{{}^{\circ}}$$

**FIGURE 3:**
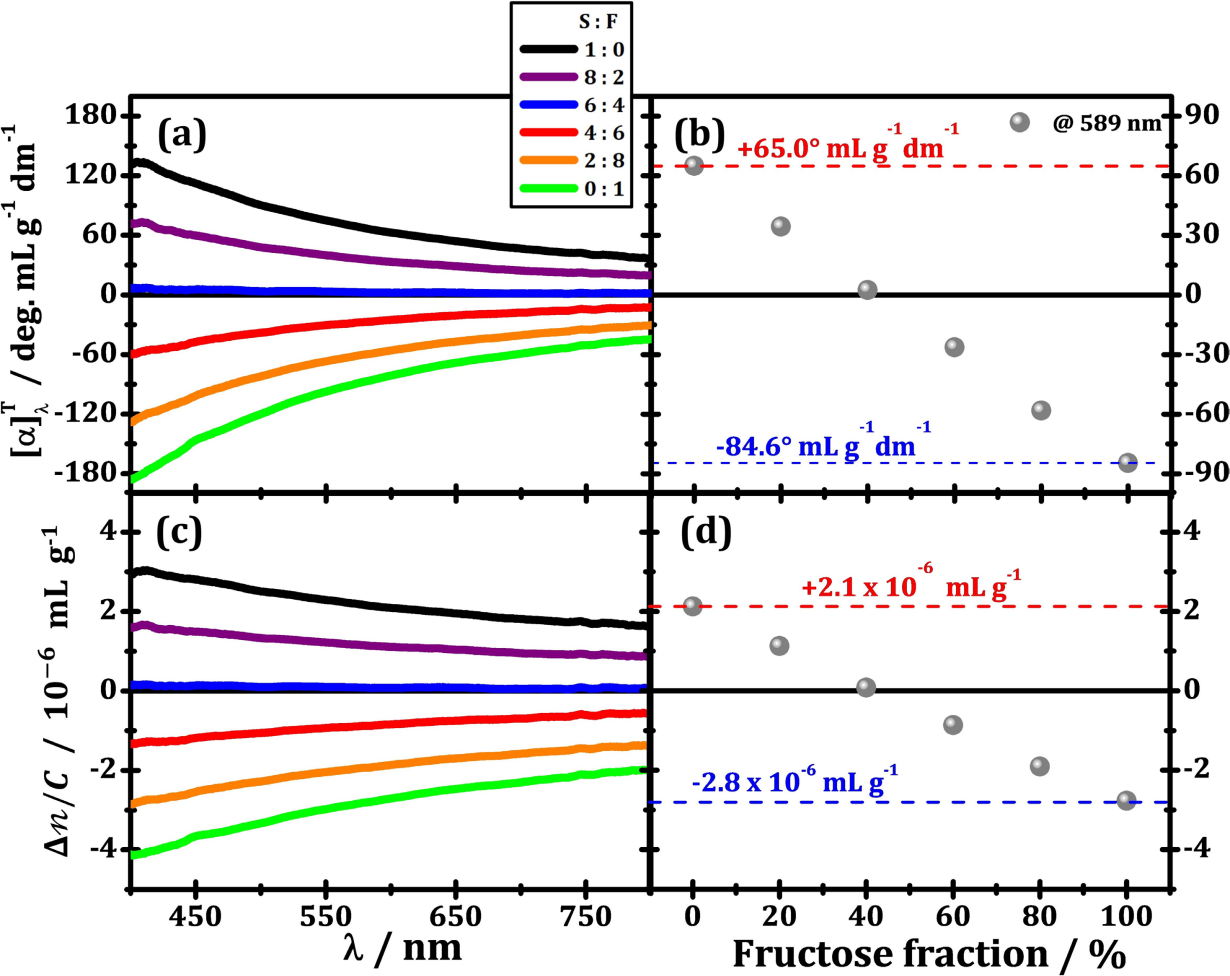
Results for specific optical rotation dispersion and specific circular birefringences over wavelength (a, c) and over fructose fraction (b, d) presented in mixtures between sucrose and fructose, that is, species of opposing chiralities.

In Figure 3a,c, respectively, the results of SORD (${\left[\alpha \right]}_{\lambda }^{T}$) and specific CB (Δn/C) as a function of wavelength are presented for six different mixed solutions, and Figure 3b,d presents SORD and CB results as a function of the fructose fraction at 589 nm. The SORD curves (Figure 3a) show an inverse dependence on wavelength, which is theoretically associated with a sum of terms inversely proportional to the wavelength squared ($\sim \lambda^{-2}$) [10, 21, 47], and a neat progression from pure sucrose's dispersion (black curve) toward pure fructose's dispersion (green curve).

Notice that for the 60% sucrose to 40% fructose mixture solution (blue curve), the overall SORD value is nearly zero throughout the spectrum, which means the optical rotation caused by three parts of sucrose cancels out the optical rotation from two parts of fructose. This observation is expected since the sucrose's rotatory power is weaker than that for fructose's, as discussed in Section 4A. The result is confirmed after finding the ratio ${\left[\alpha_{F}\right]}_{589}^{20{}^{\circ}C}/{\left[\alpha_{S}\right]}_{589}^{20{}^{\circ}C}\approx 1.4$, when using data from reference [46], which is close enough to 1.5.

The effect of increasing the fructose fraction in the mixed solution on optical rotation is more clearly seen in Figure 3b. The SOR at 589 nm is shown to evolve linearly with the fructose fraction, as suggested by Equation (16). Since $\left[\alpha_{S}\right]>\left[\alpha_{F}\right]$, the ${\left[\alpha_{\mathrm{net}}\right]}_{\lambda }^{T}$ is then a decreasing straight line, as observed in Figure 3. We highlight the values of SOR values at 0% (100%) and 100% (0%) fructose (sucrose), which are in good agreement with previous measurements [16], demonstrating that our model predicts the measured experimental behavior quite satisfactorily.

A similar trend is shown in Figure 3c,d on specific circular birefringence curves, as the wavelength‐dependence remains inverse, but with smoother rate of change ($\sim \lambda^{-1}$). The overall curves are updated proportionally to the fractional presence of fructose, as the black curves (0% fructose) linearly evolve to the green curves (100% fructose). The evolution at 589 nm is shown in Figure 3d, and we highlight the specific birefringence values for 100% sucrose and 100% fructose solutions. Fructose is shown to be more birefringent than sucrose, although with opposite chirality. The specific circular birefringence values when compared with the LB of tapes [27] are found to be three orders of magnitude weaker. This result agrees with the fact that circular anisotropies are rather subtle. The alternative analysis of circular retardance dispersion and its progression with the proportion of each chiral species in the mixed solution is displayed in Figure S4.

## 5 Discussion

By making use of novel technical advancements in spectropolarimetry, this contribution is providing a descriptive investigation of a particular application of transmission spectropolarimetry with the rotating‐retarder method, recently improved to avoid common artifacts created by non‐ideal achromatic elements [25]. Similarly to previous work [27], three limiting facts in both the model and the approach presented here should be noted: the presence of absorption, spatial heterogeneity, and temporal dynamics.

Specifically, Equations (1a) and (1b) assume a *transparent* circularly birefringent medium, wherein only the effect of birefringence upon circular components of light is considered. Adding the presence of light attenuation by considering a difference in absorption of circular components, also known as *circular dichroism*, would modify the transformation in Equations (5a)–(5d), and consequently the medium's associated Mueller matrix.

Moreover, spatial homogeneity is another assumption of this work. The solutions are considered homogeneous, having equal effect on all points of the light beam's planes transverse to the propagation. If different points of the beam experience, for instance, a gradient in concentration, with higher density of molecules on the bottom due to gravitational pull, the detected transmitted beam would yield a mixed optical rotation effect. Different light rays of a particular wavelength rotating by different amounts would contribute to the lowering of the detected degree of polarization. This effect can be avoided by properly adjusting concentrations below saturation.

Furthermore, any system containing time‐dependent optical activity should be taken with caution before analyzed with the proposed approach. It is paramount, however, to initially evaluate the time frame of the measurement of a single ORD with the inverse rate with which the signal changes. For instance, if measuring ORD takes 3 min, but ORD changes less than 10% within an hour, using this approach should be good enough to track the kinetics. Otherwise, if the signal substantially changes within a measurement duration, this methodology should be avoided or adapted. Recently, Vala et al. [26] have explored a powerful tool to study time‐resolved chiroptical properties with Mueller matrix spectroscopy. This method should be considered a step further from the simpler but still cost‐effective approach developed in the present text, where no automation is necessary. Similarly, Cortés et al. [21] have used Mueller polarimetry to study the optical activity of a quartz crystal as a study case but lacked fast spectral acquisition for proper dispersion analysis. Instead, they have made use of laser lines at different wavelengths, then fitted the optical rotation experimental data with a Lorentz model to obtain the dispersion profile. Here instead, the shape of the dispersion is determined experimentally with the CCD of a portable spectrometer, making assumptions about the theoretical fit equations unnecessary for purposes of dispersion evaluation.

Additionally, due to its simplicity, our approach can be easily associated with theoretical simulations based on density functional theory (DFT), or even machine learning techniques [48]. This synergistic approach provides a trustworthy investigative methodology to explore the relationship between chirality, defined by the global molecular arrangement of stereoisomers [4, 11], and their optical rotatory effect.

## 6 Conclusion

This work reports the use of transmission SSP to investigate circular birefringence phenomena through a simple case study to demonstrate the robustness of the approach toward optical anisotropies. Here the ORD caused by sucrose, a dextrorotatory disaccharide obtained from table sugar, and by fructose, a levorotatory monosaccharide commonly used to substitute sucrose in the food industry, are measured as a function of (1) the concentration of their aqueous solutions and (2) of their fractional contribution in a fructose/sucrose mixture. The results are corroborated by the presence of a linear trend theoretically expected from both scenarios, and by the form of the dispersion curves, allowing for an inverse wavelength behavior. Our spectropolarimeter is capable of measuring beyond only the wavelength‐resolved rotation of plane‐polarized light (e.g., changes in ellipticity or degree of polarization). In fact, its application in case studies like the ones present here is a recognition of its versatility and power as an alternative tool to analyze more complex effects such as SORD. As a proof‐of‐concept, the academic community shall find value in seeing a theoretical framework being used to analyze a historically important chiroptical effect with an improved experimental approach. The range of applicability of such approach, however, is expected to reach larger areas involved in chiroptical spectroscopy, such as biochemistry and organic electronics, especially when induced extrinsic stimuli, for example, magnetic fields, are present. This contribution also provides an analysis simple enough to be carried out as a pedagogical approach to introduce students into the world of either polarization optics or stereochemistry.

## Supporting information


**Data S1** Supplementary Information.

**Figure S1** Scheme to represent the effect of a circularly birefringent sample on polarized light.

**Figure S2** Plots of optical rotation (ΔΨ in degrees) as a function of concentration (in $g\;mL^{-1}$) for (a) sucrose and (b) fructose evaluated at sodium D‐line (589 nm) for reference. The red lines are linear fit curves with slope as the only free parameter (y‐axis intercept parameter fixed at zero). The respective fit results are displayed as insets.

**Figure S3** Spectra of circular retardance $\Upsilon_{0}$ for aqueous solutions of (a) sucrose and (b) fructose over a range of concentration and specific circular retardance $\left[\Upsilon_{0}\right]$ of (c) sucrose and (d) fructose.

**Figure S4** Specific circular retardance $\left[\Upsilon_{0}\right]$ from aqueous mixed solutions of sucrose and fructose, both with concentration of 0.1 g/mL, in different volumetric proportions, specified in the inset label.

## Data Availability

Data may be made available upon request.
