# Supplementary material for: Stokes Spectropolarimetry Applied to Measure Circular Birefringence Dispersion of Aqueous Solutions of Sugars
Source: Chirality. 2025 Jul 8;37(7):e70047. doi: 10.1002/chir.70047 (PMC12238699; doi:10.1002/chir.70047)
Supplement: Supplementary file 1 — Data S1 Supplementary Information. [file CHIR-37-e70047-s003.docx]

**SUPPLEMENTARY MATERIAL FOR:**

**Stokes spectropolarimetry applied to measure circular birefringence dispersion of aqueous solutions of sugars**

**Ruan Lucas Sousa Lima^1, *^, Eric Santos da Silva,^1^ Paulo Trindade Araujo, ^2, ¶^ & Newton Martins Barbosa Neto^1^**

¹Institute of Natural Sciences, Federal University of Pará, Belém, Pará 66075-110, Brazil

²Department of Astronomy and Physics, The University of Alabama, Tuscaloosa, Alabama 35487-0324, USA

^*^ ruan.lima@icen.ufpa.br (corresponding author)

^¶^ paulo.t.araujo@ua.edu

**S1. PHENOMENOLOGICAL CIRCULAR BIREFRINGENCE THEORY**

This section aims to portray the basic phenomenological theory behind circular birefringence. A similar description is present in previous work on linear birefringence phenomena [1]. Here, the circular birefringence effect (CB) is depicted in **Figure S1**, as the total phase difference, or *circular retardance,* between right and left-handed components of light is taken here to be $\Upsilon_{0}$. For symmetry reasons, we shall consider $\pm\Upsilon_{0}/2$ to be the induced phase for each component, respectively, such that  [2,3]

$\Upsilon_{0}\left( \mathrm{waves} \right)={\Upsilon_{0}\left( \mathrm{rad} \right)}/{2\pi}= L \Delta\mathcal{n /}\lambda$ , **(S1)**

where $\Delta\mathcal{n=}\mathcal{n}_{\mathcal{L}}-\mathcal{n}_{\mathcal{R}}$ is the circular birefringence of the medium, $L$ is the crossed path length, and $\lambda$ is the wavelength of the analyzed beam.


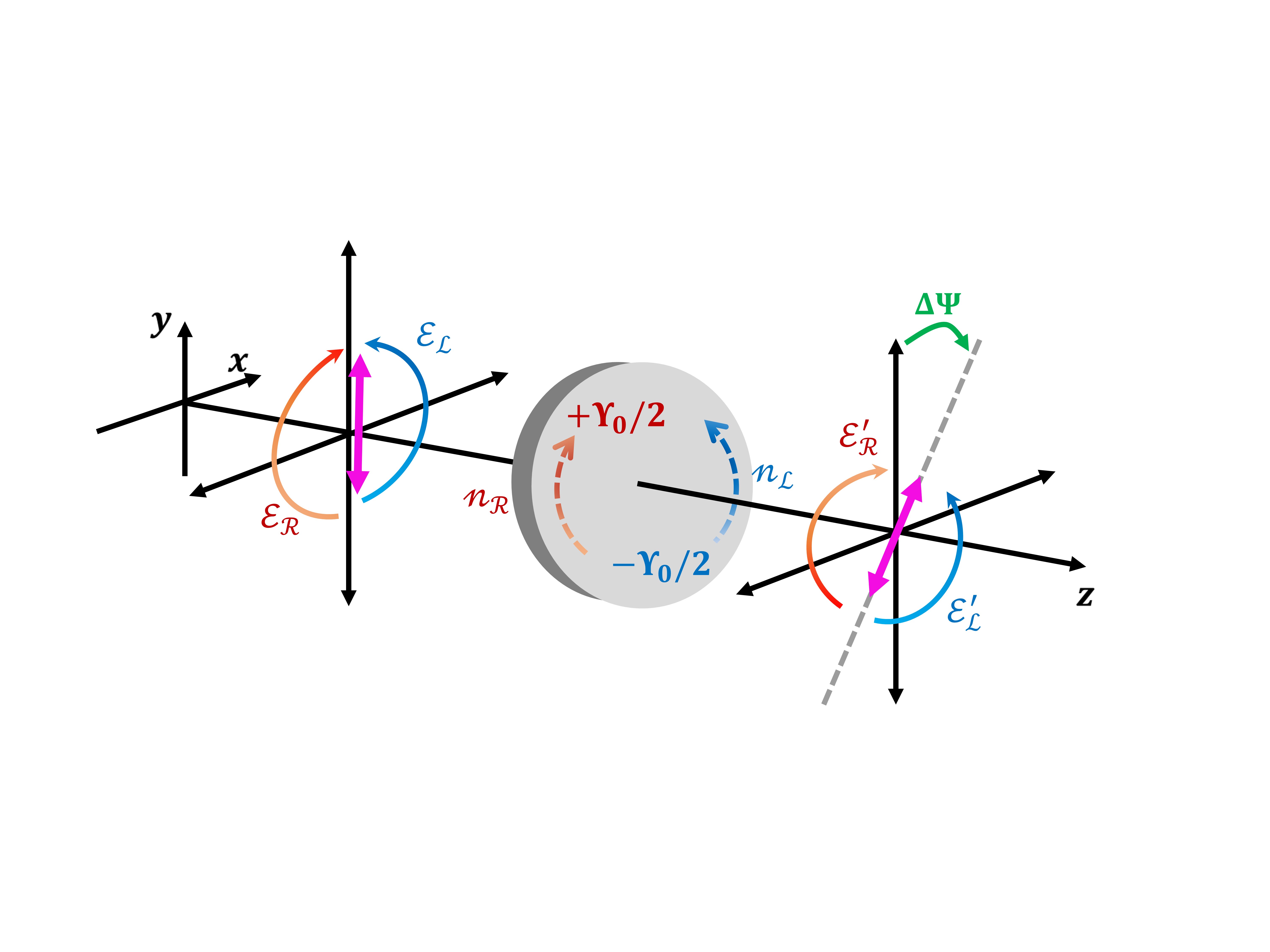


Figure S1: Scheme to represent the effect of a circularly birefringent sample on polarized light.

The net effect of a circular birefringent medium is then to rotate the plane of polarization of incident light, as shown in the next section. This effect is caused by some molecules possessing *chirality*, defined as the property of having non-superimposable mirror images  [4,5]. Chirality is important if, and only if, two chiral species interact  [6]. This mirror asymmetry implies a preferential direction in the helical interaction of light with chiral molecules, which in the case of non-resonant interaction, means each circular component experiences a different speed of propagation.

Due to rotational symmetry of the circular components around the propagation axis, CB is expected to be independent on angular position of the sample, especially when diluted in solution. This birefringence-induced rotation is then measured as a change of inclination of the ellipse of polarization, which can either be a clockwise (for *dextrorotatory* molecules, like sucrose) or counterclockwise (for *levorotatory* molecules, like fructose*)* optical rotation. The amount of rotation per unit length crossed by light inside the chiral medium is called *rotatory power*, and is empirically given by  [3]

${\Delta\Psi}/L=\left[ \alpha\right]_{\lambda}^{T} C$ , **(S2)**

where $\Delta\Psi$ is the optical rotation (difference in elliptical inclination) in degrees, $L$ is the optical path length crossed by light inside the birefringent medium in decimeters ($dm$), C is the concentration of the solution in $g/mL$, and $\left[ \alpha\right]_{\lambda}^{T}$ is the *specific optical rotation* (SOR) at wavelength $\lambda$ and temperature $T$. The wavelength-dependence of SOR is called *specific optical rotation dispersion* (SORD) and is an intrinsic characteristic of each optical active material, given a temperature and solvent.

Thus, the SORD establishes the linear proportionality between rotatory power and concentration for each chiral species and hence can be obtained by a linear regression of rotatory power data over different concentrations of each sample. Here, we use sucrose, fructose, and mixtures thereof, as a proof of concept for the connection between Stokes parameters and circular birefringence (through SORD). By measuring wavelength-dependence of $\mathbb{S}_{1}$ and $\mathbb{S}_{2}$ for transmitted light, we calculate $\Psi$ dispersion, compare it with a reference (solvent-only reference) $\Psi_{0}$, and determine the optical activity of chiral organic compounds. In the following sections a model to connect Mueller matrices, Stokes parameters, SORD and circular birefringence is derived and analyzed.

**S2. OPTICAL ROTATION DATA AT 589 NM**

**
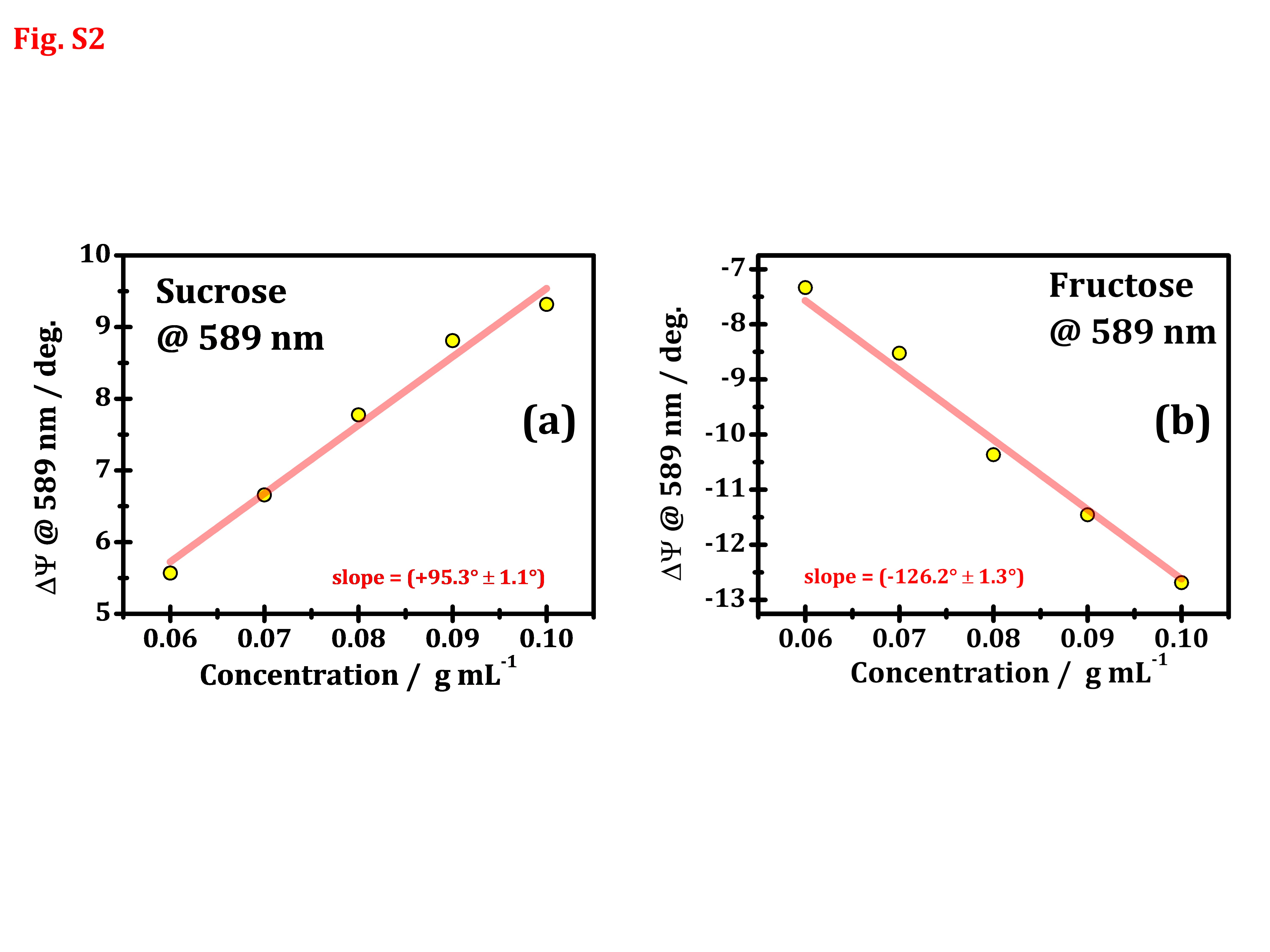
**

**Figure S2:** Plots of optical rotation ($\Delta\Psi$ in degrees) as a function of concentration (in $g mL^{-1}$) for (a) sucrose and (b) fructose evaluated at sodium D-line (589 nm) for reference. The red lines are linear fit curves with slope as the only free parameter (y-axis intercept parameter fixed at zero). The respective fit results are displayed as insets.

**S3. CIRCULAR RETARDANCE DISPERSION**

In this manuscript, circular birefringence has been largely discussed upon its consequences as optical rotation, usually quantified as rotatory power (rotation per unit length) or specific optical rotatory dispersion (rotation per unit length per unit concentration as a function of wavelength). Here follows an alternative unusual discussion on circular retardance dispersion, as a comparison to a previous work using the same methodology to study linear retardance  [1].

In **Figures S3(a)** and **S3(b)**, the dispersions of circular retardance of aqueous solutions of sucrose and fructose are displayed for various concentrations. By making use of **Equation (10)** in the main part of the paper, each curve was obtained by multiplying the optical rotation dispersion ($\Delta\Psi(\lambda)$) curves in **Figure 2(a) and 2(b)**, which yields a retardance in units of degrees, and then dividing by 360° (reference for a whole wavelength) to convert it into units of wavelengths, as given in **Equation (S1).**


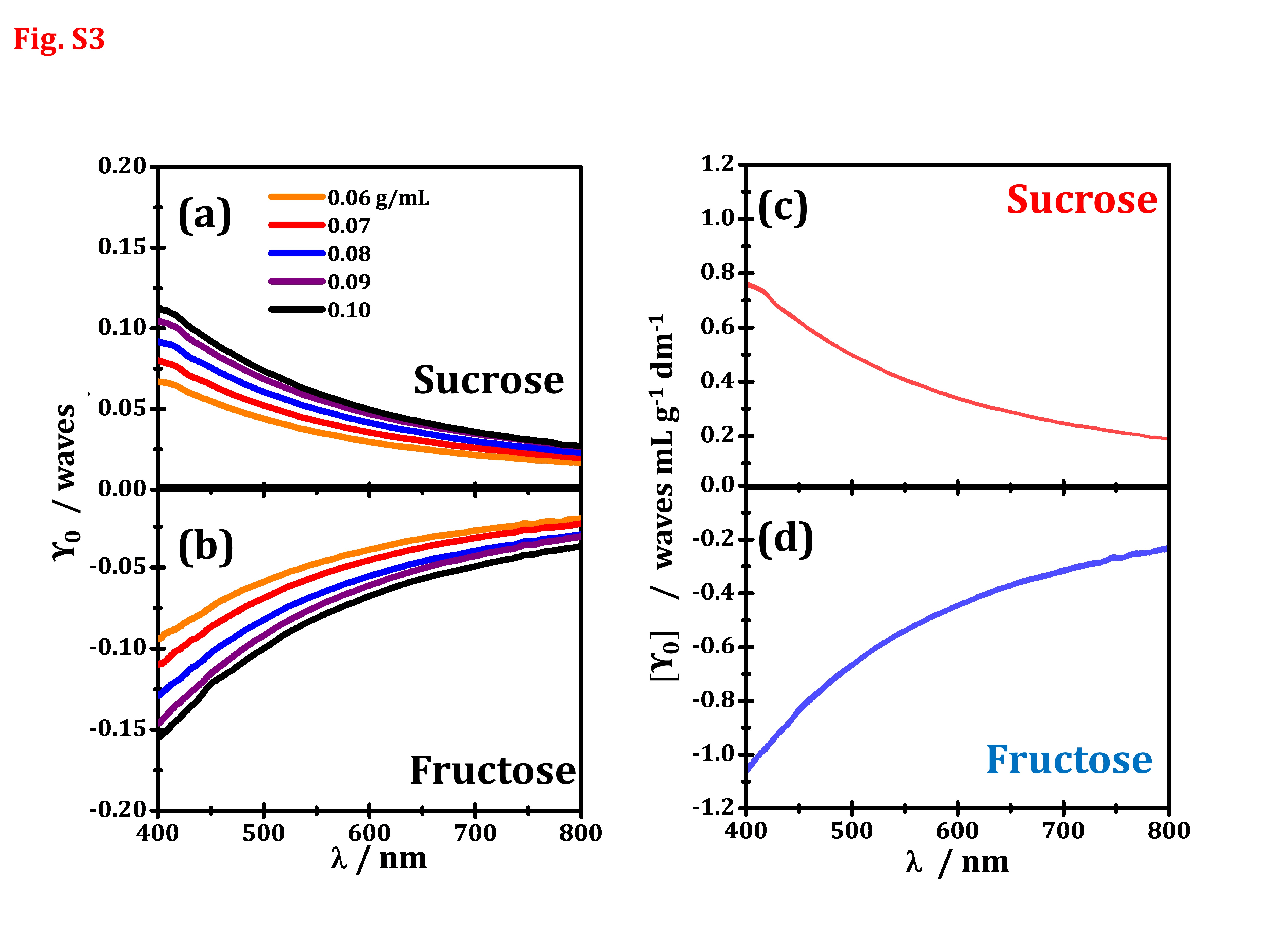
Now, by dividing each curve of **Figures S3(a)** by the solution cell optical path length $(L=1.5 dm$) and then by its associated concentration value (the orange curve would be divided by 0.06, for example), all the curves would fall into a similar dispersion of *specific circular retardance* ($[\Upsilon_{0}]$, circular retardance per unit length per unit concentration), displayed in Figure S3(c). The fructose counterpart is shown in Figure S3(d). In these plots, the red and blue curves, representing the specific circular retardance of sucrose and fructose, respectively, were obtained by taking the arithmetic average of the specific circular retardance curves obtained from each concentration and extracting the standard deviation. The thickness of the lines follows the size of the error bar at each detected wavelength.

**Figure S3:** Spectra of circular retardance $\Upsilon_{0}$ for aqueous solutions of (a) sucrose and (b) fructose over a range of concentration and specific circular retardance $\left[ \Upsilon_{0} \right]$ of (c) sucrose and (d) fructose.

Similarly, in **Figure S4**, the specific circular retardance dispersion of the mixed solutions are displayed for each volumetric proportion between sucrose and fructose. Analogously to the linear retardance generated by birefringent adhesive tapes and their linear progression with the number of layers stacked [1], the circular retardance generated by birefringent sugar solutions can be tuned with the appropriate proportion and concentration of chiral species.


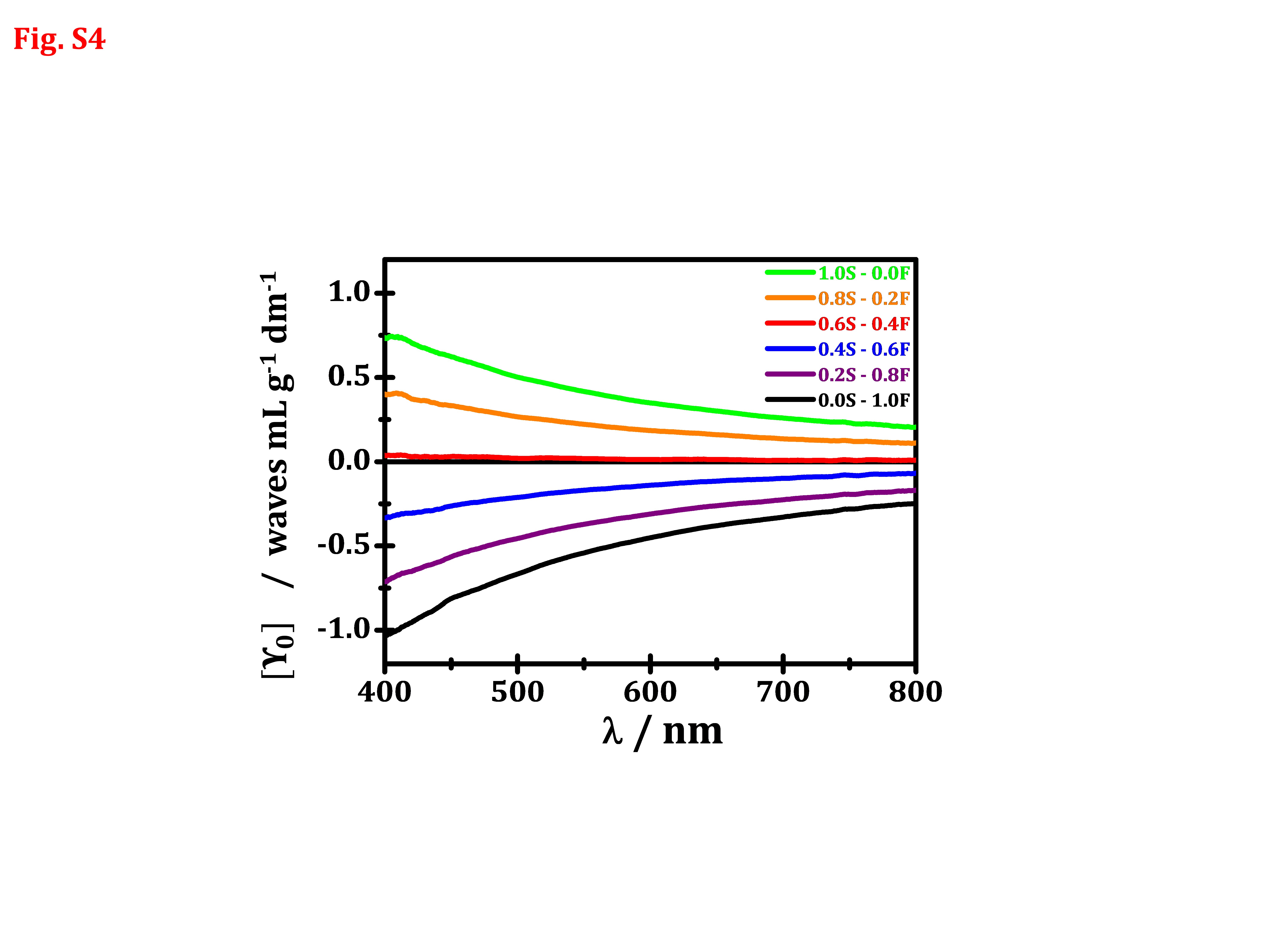


**Figure S4:** Specific circular retardance $\left[ \Upsilon_{0} \right]$ from aqueous mixed solutions of sucrose and fructose, both with concentration of 0.1 g/mL, in different volumetric proportions, specified in the inset label.

**REFERENCES**

1. R. L. S. Lima, E. S. Silva, P. T. Araujo, and N. M. Barbosa Neto, "Measuring linear birefringence via rotating-sample transmission Stokes spectropolarimetry," Appl Opt **63**, 7625 (2024).

2. D. H. Goldstein, *Polarized Light* (CRC Press, 2017).

3. E. Hecht, *Optics*, 5th ed. (Pearson, 2017).

4. D. M. Bishop, *Group Theory and Chemistry* (The Clarendon Press, 1973).

5. K. Mislow, *Introduction to Stereochemistry* (Dover publications, 2002).

6. B. Nordén, A. Rodger, and T. Dafforn, *Linear Dichroism and Circular Dichroism: A Textbook on Polarized-Light Spectroscopy* (RSC Publishing, 2010).
