# Supplementary figures and images for: Stokes Spectropolarimetry Applied to Measure Circular Birefringence Dispersion of Aqueous Solutions of Sugars
Source: Chirality. 2025 Jul 8;37(7):e70047. doi: 10.1002/chir.70047 (PMC12238699; doi:10.1002/chir.70047)

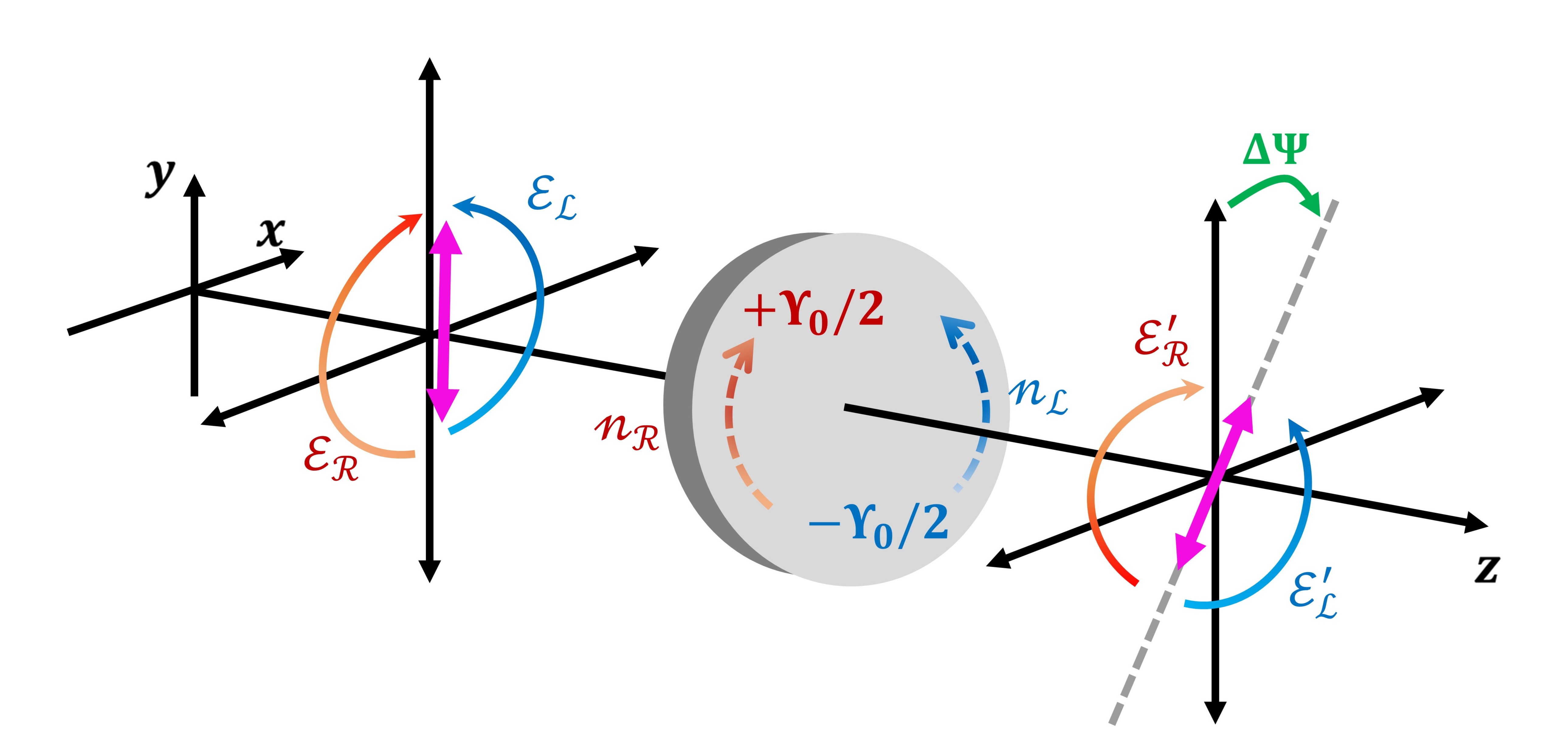

Supplement: Supplementary file 2 — Figure S1 Scheme to represent the effect of a circularly birefringent sample on polarized light. [file CHIR-37-e70047-s001.jpg]

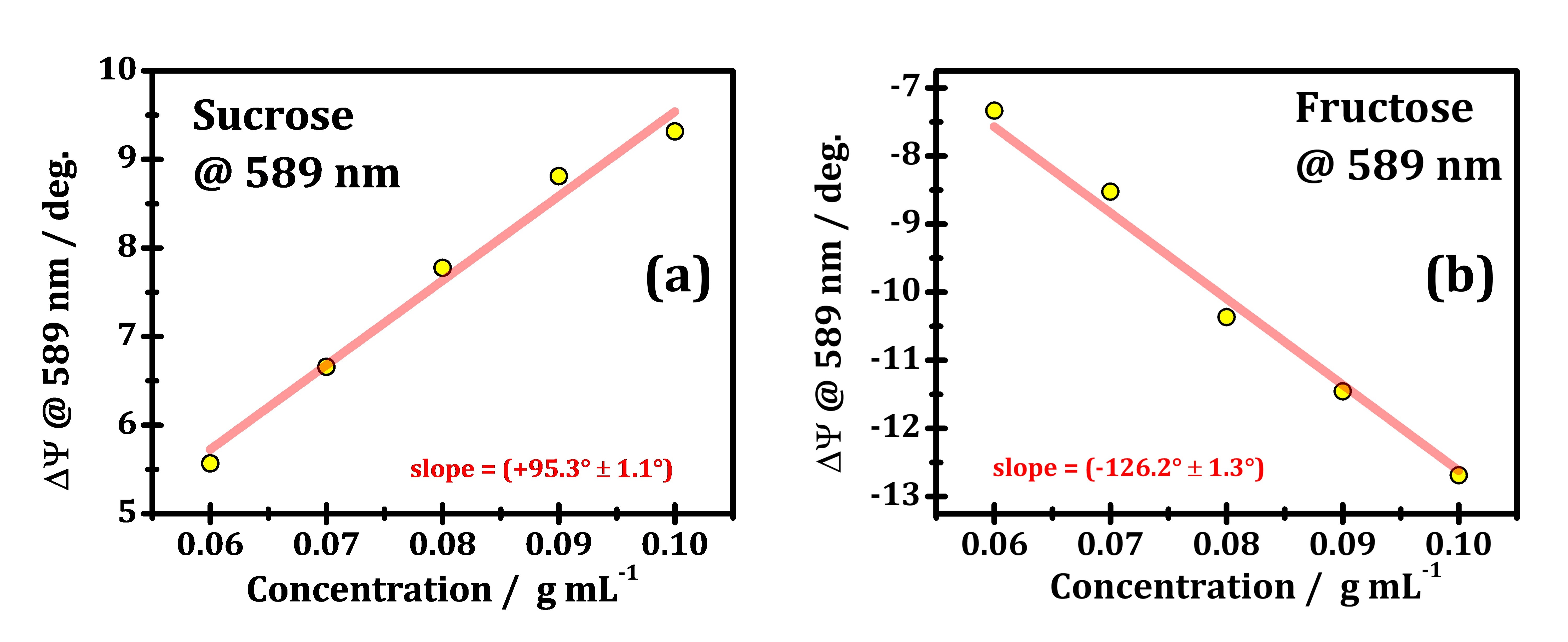

Supplement: Supplementary file 3 — Figure S2 Plots of optical rotation (ΔΨ in degrees) as a function of concentration (in gmL−1) for (a) sucrose and (b) fructose evaluated at sodium D‐line (589 nm) for reference. The red lines are linear fit curves with slope as the only free parameter (y‐axis intercept parameter fixed at zero). The respective fit results are displayed as insets. [file CHIR-37-e70047-s002.jpg]

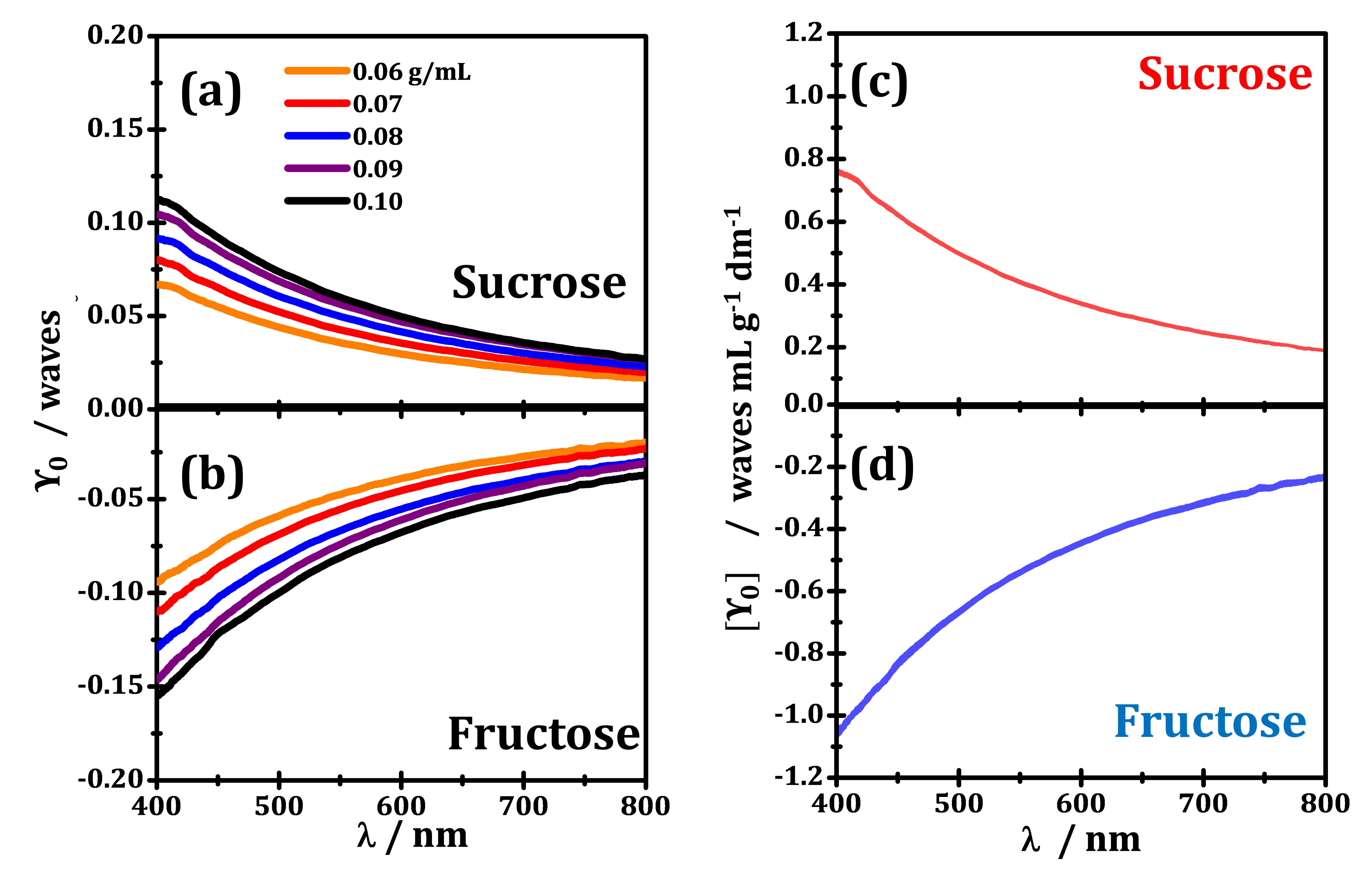

Supplement: Supplementary file 4 — Figure S3 Spectra of circular retardance Υ0 for aqueous solutions of (a) sucrose and (b) fructose over a range of concentration and specific circular retardance Υ0 of (c) sucrose and (d) fructose. [file CHIR-37-e70047-s005.jpg]

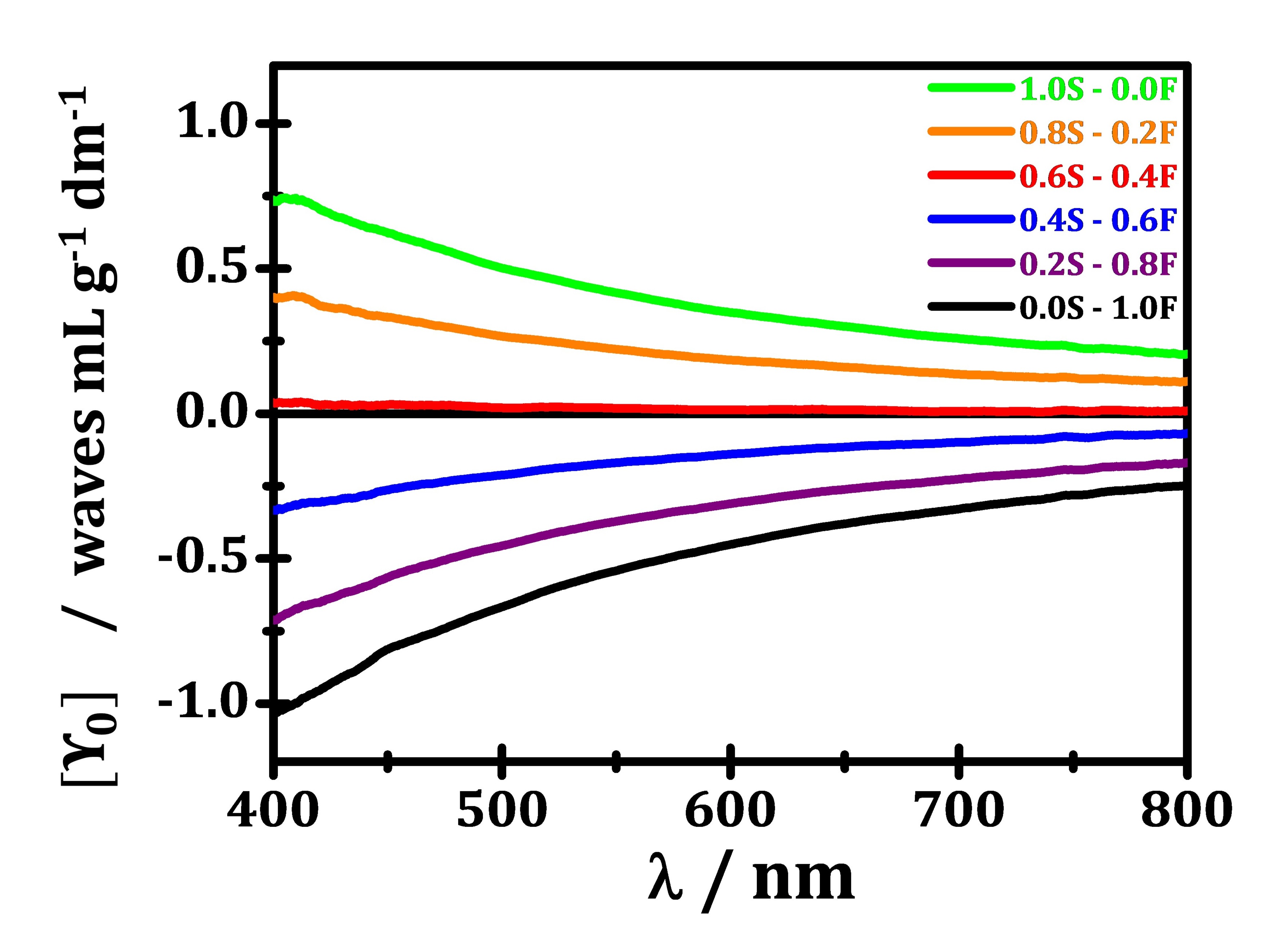

Supplement: Supplementary file 5 — Figure S4 Specific circular retardance Υ0 from aqueous mixed solutions of sucrose and fructose, both with concentration of 0.1 g/mL, in different volumetric proportions, specified in the inset label. [file CHIR-37-e70047-s004.jpg]
